# Evaluation of modelled net primary production using MODIS and landsat satellite data fusion

**DOI:** 10.1186/s13021-016-0049-6

**Published:** 2016-06-02

**Authors:** Steven Jay, Christopher Potter, Robert Crabtree, Vanessa Genovese, Daniel J. Weiss, Maggi Kraft

**Affiliations:** 1 Yellowstone Ecological Research Center, 2048 Analysis Dr. Ste. B, Bozeman, MT 59718 USA; 2 CASA Systems 2100, LLC, PO Box 1631, Los Gatos, CA 95030 USA; 3 Science and Environmental Policy, California State University, Monterey Bay, 100 Campus Center, Seaside, CA 93955 USA; 4 Department of Zoology, University of Oxford, The Tinbergen Building, S Parks Rd, Oxford, OX1 3PS UK

**Keywords:** Net primary production, MODIS, Landsat, EVI, Ameriflux

## Abstract

**Background:**

To improve estimates of net primary production for terrestrial ecosystems of the continental United States, we evaluated a new image fusion technique to incorporate high resolution Landsat land cover data into a modified version of the CASA ecosystem model. The proportion of each Landsat land cover type within each 0.004 degree resolution CASA pixel was used to influence the ecosystem model result by a pure-pixel interpolation method.

**Results:**

Seventeen Ameriflux tower flux records spread across the country were combined to evaluate monthly NPP estimates from the modified CASA model. Monthly measured NPP data values plotted against the revised CASA model outputs resulted in an overall R^2^ of 0.72, mainly due to cropland locations where irrigation and crop rotation were not accounted for by the CASA model. When managed and disturbed locations are removed from the validation, the R^2^ increases to 0.82.

**Conclusions:**

The revised CASA model with pure-pixel interpolated vegetation index performed well at tower sites where vegetation was not manipulated or managed and had not been recently disturbed. Tower locations that showed relatively low correlations with CASA-estimated NPP were regularly disturbed by either human or natural forces.

## Background

Net photosynthetic accumulation of carbon by plants, also known as net primary production (NPP), captures solar energy and drives most biotic processes on Earth. Climate controls on NPP fluxes on land are an issue of central relevance to humanity, in part due to possible limitations on the extent to which NPP in managed ecosystems can provide adequate food and fiber for growing populations [26].

Measurement of NPP presents many challenges in any ecosystem, and particularly in heterogeneous environments such as wetland, cultivated, ex-urban, and mountainous landscapes. Traditionally, NPP has been calculated by harvesting and measuring dry biomass or from eddy flux towers estimates [35]. Measuring dry biomass is labor- and time-intensive and logistically impossible to perform at scales other than the small plot (generally < 1 ha) [14]. Eddy flux towers measure the amount of CO_2_ being exchanged with the atmosphere across a landscape. This technique can cover a larger area than using small plot biomass measurements. However, eddy flux tower measurements are affected by wind direction and atmospheric conditions, and logistical limitations have led to under-representation of tower sites in remote, disturbed, or degraded ecosystems [8].

Advances in modeling techniques and the integration of satellite multi-spectral data can greatly increase our capacity to estimate global and continental NPP. Common gridded approaches using satellite imagery to estimate NPP have assumed a constant land cover type within each pixel [10, 20, 32, 43]. By making this generalization, the influence of land cover types covering small fractions of a pixel is potentially lost when pixels are classified. This can have detrimental effects on the pixel’s estimate of NPP if some highly productive systems such as wetlands, or conversely, low productive areas such as bare ground, are ignored. In an attempt to improve NPP estimates across North America, we have developed an image fusion technique to incorporate high resolution land cover data into a modified version of the Carnegie Ames Stanford Approach (CASA) ecosystem model [20, 26] to improve NPP estimates at a 0.004 degree resolution.

One widely used estimate of global productivity is the MODIS MOD17 algorithm [43]. The MODIS MOD17 product currently estimates gross primary production (GPP) and NPP as a fraction of GPP at a 1-km spatial scale and an 8-day temporal scale. Turner et al. [36] found generally strong agreement of ground based measurements with MODIS productivity products using the BigFoot [34, 35] validation procedure. However, the MODIS products tended to overestimate NPP at low productivity sites and underestimated NPP at highly productive sites outside the tropical zone [43]. This overestimation has been mostly attributed to high light interception values during the annual maxima as well as anomalous values in non-growing seasons. The low estimation tended to be a function of using incorrect light use efficiency terms for NPP [36].

The CASA ecosystem model [20, 26] uses freely available geographic data layers for climate and soils, and satellite imagery to estimate monthly NPP. CASA has been applied and tested around the world in hundreds of published studies (e.g., [2, 7, 9, 11, 18]). The CASA model estimates NPP at optimal metabolic rates, adjusting these rates based on scalars related to the effects of climate, soil moisture and texture, and land use [20]. For this study, CASA NPP is calculated based on a constant maximum light use efficiency concept [15], the MODIS vegetation index, solar radiation, temperature, and a soil moisture scalar.

Previous NPP estimates using the CASA model have shown strong correlation in timing with eddy flux towers; Potter et al. [26] reported an *R* ^2^ = 0.77 using a small set of monthly AmeriFlux tower NPP estimates. The CASA model was also capable of predicting annually summed NPP with an *R* ^2^ = 0.90 on a global scale using the NOAA Advanced Very High Resolution Radiometer (AVHRR) VI and regressing against over 1900 observed NPP data points [22]. The model has been used successfully in measuring the global effects of deforestation on NPP [21, 26], particularly in tropical Amazonian and Asian carbon fluxes [23, 27, 41].

We have developed a new approach for this study to improve the spatial resolution and potentially the accuracy of the CASA NPP model by using an image fusion technique, whereby high resolution, Landsat-derived land cover is fused with MODIS MOD13A1 Enhanced Vegetation Index (EVI) data. The proportion of each land cover type within each pixel area was used to influence the ecosystem model result. This technique produced CASA model predictions of monthly NPP at 0.004 degree (approx. 500-m) resolution for North America from 2000–2010 that were compared to tower flux estimates of NPP for evaluation.

## Methods

The CASA model requires several input data sets in order to successfully estimate NPP values. Land cover specific interpolated vegetation indices, gridded temperature and precipitation data, soil texture, solar radiation, and elevation are all used to estimate NPP. The vegetation indices, climate data, and solar radiation datasets were compiled for each month from 1999 to 2010 and the soil texture and elevation data remains static. These data parameterize the CASA model which is programmed in Python and integrates with ArcGIS software using the ArcPy processing module. Data inputs and CASA processing are described in more detail below.

### Interpolated vegetation index

**Fig. 1:**
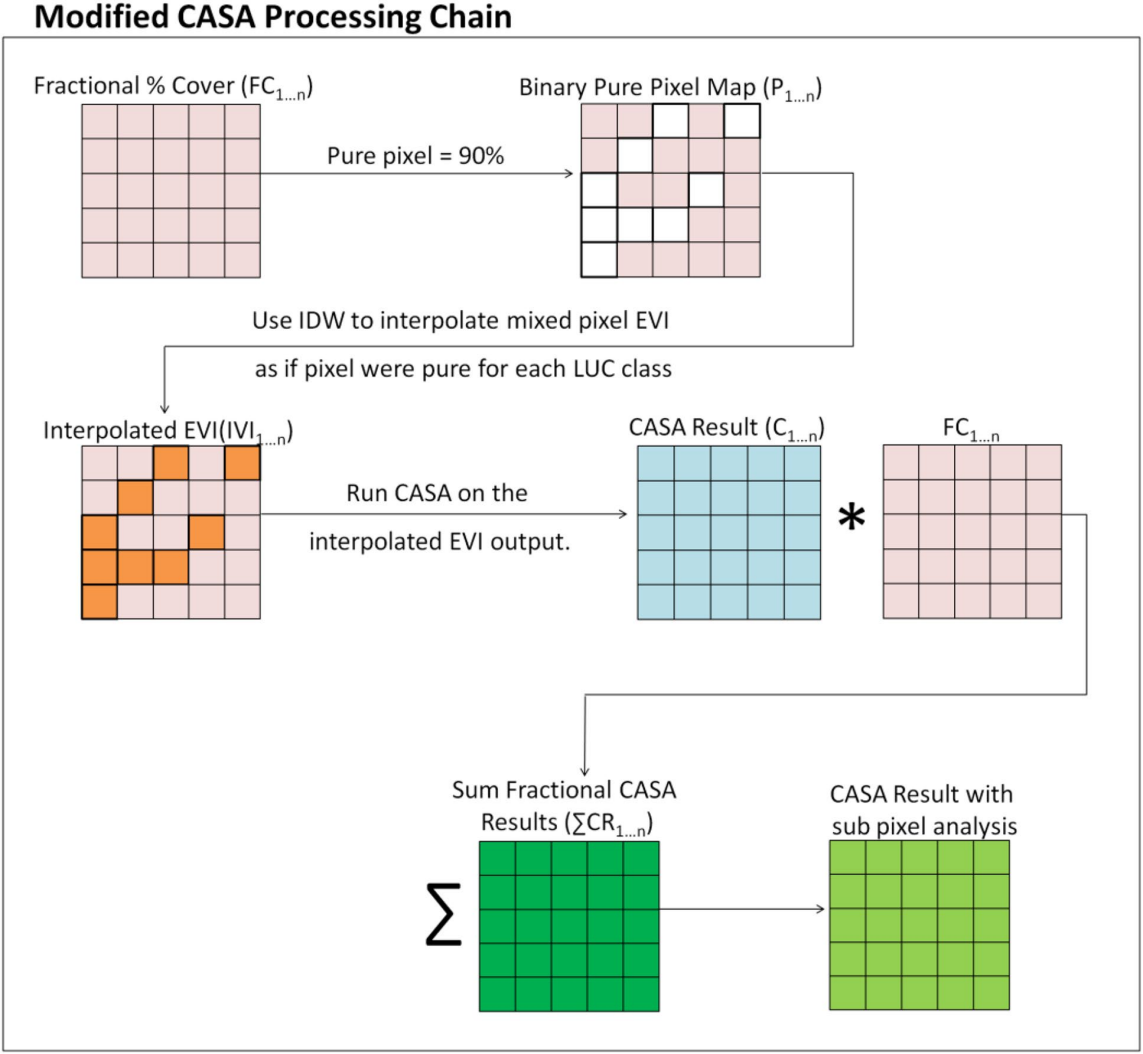
Processing steps for the modified CASA algorithm. (1) Identify 500-m MODIS pixels containing >90 % cover of the same NLCD land cover class. (2) Using pure-pixels identified in step 1 as points, interpolate (IDW) mixed pixel EVI values as if the interpolated cell were pure for each cover class. (3) Run CASA for all cover types using the corresponding pure-pixel interpolated EVI raster developed in step two. (4) Multiply the CASA outputs by the fractional % cover raster. (5) Sum the outputs from step 4

An interpolated vegetation index was created using a combination of MODIS MOD13A1 16-day EVI data and both 2006 NLCD [3] land cover data and Canadian land cover data [6, 12, 17]. A five step process was developed to create land cover specific CASA NPP estimates, which could then be aggregated to estimate total landscape NPP (Fig. 1).

Step one of the processing chain created “fractional cover” estimates. Fractional cover estimates were created by combining monthly 0.004 degree resolution, cloud-filled [40] MODIS EVI data with 0.0003 degree (approx. 30-m) resolution 2006 NLCD and 2001 Canadian land cover data to estimate the proportion of each land cover contained in a MODIS pixel. High resolution land cover data was aggregated to 11 general land cover classes. The 11 classes used in modeling were; water, evergreen forest, wetlands, lichen, mixed forest, woodlands, grasslands, croplands, deciduous forest, and brushland. The high resolution land cover data was then resampled to 0.004 degree resolution, while maintaining the proportion of each land cover type contained within the pixel to create a fractional cover layer.

The next step required the identification of “pure-pixels”. Pure pixels are pixels that contain more than 90 % of a single class. These pure pixels are used to extract MODIS EVI values using a point-intercept method. Pure pixels are identified by applying a mask to each fractional land cover data set and remove all pixels with a value below 0.9. These pixels are then converted to a point representing the center of its corresponding pixel. These points a then overlaid with the monthly MODIS EVI data and the intersecting pixel values are extracted for each month from 2000 to 2010.

The third step requires using the pure pixel values to produce a land cover specific vegetation index by performing an inverse distance weighted (IDW) algorithm. The land cover specific pure pixels tended to be highly clustered making the IDW method appropriate because of Tobler’s Law [33]. The IDW process was run using ESRI ArcGIS 10.2 software. The optimal power function for each land cover was calculated using the Geostatistical Analyst extension in ArcGIS 10.2. The optimal power function is identified using cross-validation to find a minimum root mean square prediction (RMSPE) value [19, 39] and a variable search radius was used.

Next, the interpolated vegetation indices (IVI) are used as the vegetation input for the CASA model. The CASA model uses the IVI along with solar radiation and temperature and wetness scalars to estimate NPP [20]. Each land cover is run independently through the CASA model to produce NPP estimates specific for each cover type. These results are then multiplied by the corresponding fractional cover value calculated in step 1. This step produces NPP estimates proportional to the amount of each land cover type per pixel.

In the final step, the proportional NPP estimates are summed to create a total NPP estimate per pixel. This total NPP value is now influenced by the proportion of the pixel covered by specific land cover type. This sub pixel analysis provides the opportunity to estimate NPP values without using the assumption that a MODIS pixel is a homogenous land cover as previous estimates have done [22, 26].

### Climate data

Two consecutive years of precipitation and temperature data are required for CASA model initialization in order to model the soil moisture reservoir (explained in detail below). For model years 2000 and 2001, National Centers for Environmental Prediction (NCEP) North American Regional Reanalysis (NARR) climate datasets were used as input data for the CASA model. These gridded datasets had a spatial resolution of 0.3 degree (approximately equal to 25 km resolution for the continental USA) and were developed at NOAA’s National Center for Atmospheric Research (NCAR). The NARR data is an extension of the NCEP global reanalysis. The NARR model uses the high resolution NCEP data along with an advanced data assimilation model. This assimilation technique improves the accuracy of gridded temperature and precipitation estimates. NCEP data was used for the years 2000 and 2001 because the higher resolution MODIS land surface temperature data was not available prior to 2001 and CASA initialization requires data from 1999 and 2000 to properly initialize. Model year 2001 used NCEP data so as to avoid mixing two different data sources during modeling.

For model years 2002–2010, MODIS MOD11 Terra land surface temperature data was used. The MODIS land surface temperature product is a 0.05 degree (approximately 3 km) resolution, daily global product (MOD11C1). These values were derived from the daily MODIS day/night LST/E product from pairs of 7 daytime and nighttime MODIS TIR bands (20, 22, 23, 29, and 31–33). These data inputs were used for years after 2001, because they did not exist prior for years prior to 2000. MODIS land surface data requires some pre-processing in order to obtain monthly average values. Raw data was converted from raw digital numbers to degrees Celsius by multiplying the raw value by a pre-determined scaling factor of 0.02 and subtracting 273.15 [37]. All temperature data was then resampled to 0.004 degrees for input into the CASA model.

The Global Land Data Assimilation System (GLDAS) Noah Land Surface Model L4 monthly 1.0 degree precipitation models were used as the CASA input. This 1.0 degree (approximately 75 km) resolution data set assimilates measurements from several ground-based weather stations to interpolate climatic variables. Average monthly precipitation data sets were downloaded from the NASA Goddard Earth Sciences Data and Information Services Center (http://disc.sci.gsfc.nasa.gov/services/grads-gds/gldas). GLDAS precipitation data was obtained as daily precipitation averages. To convert the data to total monthly averages, the daily average was multiplied by the number of days in its associated month. The data was then resampled to 0.004 degrees for input into the CASA model.

### Solar radiation

Monthly solar radiation values were modeled for North America. Surface solar irradiance was estimated using the ESRI ArcGIS solar radiation model. Total radiation for each pixel on a topographic surface is calculated by estimating the sum of the direct and diffuse radiation across all of North America [4, 5, 31]. Direct and diffuse radiation simulate shadow patterns at discrete intervals through time across the landscape [5].

### Soil texture and elevation

Soil texture data was obtained from the SSURGO database (NRCS) for the Continental United States and Alaska. Canadian soil texture data was obtained from the Canadian Soil Database using the Soil Landscapes of Canada (SLC) data. These datasets were than simplified into basic soil texture classes based on the content of clay, which can be interpreted by the CASA model. The seven classes are: organic soils, 0–5 % clay, 5–15 % clay, 15–30 % clay, ≥30 % clay, and lithosols. This data is resampled to 0.004 degree resolution for input into the CASA model. Elevation data was obtained from the USGS National Elevation Dataset (NED) at 0.008 degree resolution and resampled to 0.004 degree resolution for CASA model input.

### CASA ecosystem model

Monthly flux of the net fixation of CO_2_ by vegetation is computed by the CASA model using light use efficiency (LUE) [25]. Monthly NPP is estimated using surface solar irradiance, Sr, an interpolated vegetation index, a constant light use efficiency term (*e*_max_), and scalar values for temperature (T) and moisture (W). These terms are used to calculate NPP using this equation

$$NPP = Sr \times IVI \times e_{\rm max} \times T \times W$$

The *e*_max_ term is set to a constant 0.55 gC/MJ PAR, this value was determined by using predicted annual NPP values compared to previous field estimates [20, 22, 24]. Previous studies have found this value to produce good results when initializing CASA with MODIS EVI data [16, 22, 26]. The T scalar is calculated using a derivation of optimal temperatures (T_opt_) for vegetative growth. This setting varies by latitude and longitude, ranging from 0 °C in Polar Regions to 30–35 °C in low latitude deserts. Monthly water deficits define the W scalar by comparing precipitation and soil water to potential evapotranspiration (PET) using the Priestly and Taylor method [30]. CASA model initialization requires at least two consecutive years of data of vegetation data, temperature and precipitation data. The 2 years are required to model the previous years’ soil moisture reservoir and estimate the current year’s water balance.

The CASA model couples evapotranspiration to water content in the soil profile by using a series of algorithms. The model’s algorithms use three soil layers (surface organic matter, topsoil, and subsoil to rooting depth), which can have different textures, moisture holding capacity, and carbon–nitrogen dynamics [25]. These soil layers are used to calculate a water balance using precipitation and soil parameters versus evapotranspiration and drainage. Inputs from precipitation recharge the soil layers until field capacity is reached and excess is then defined as drainage and leave the system as runoff.

The CASA model is run independently on each land cover’s IVI with all other inputs remaining the same. These land cover specific modeling results are then scaled proportionally to amount of each respective land cover present in the pixel. If a particular land cover is not present in a pixel, the CASA result is not included in the final composite estimate. This ensures that only the influence of cover types present is used to calculate the final NPP value.

### NPP evaluation procedure

**Fig. 2:**
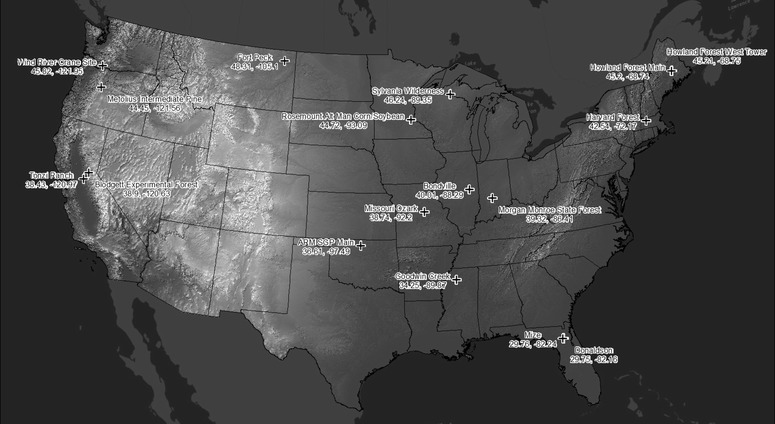
Tower flux site locations used for comparisons to CASA model NPP estimates

A total of 51 Ameriflux tower sites with over 2000 combined data points were reviewed for CASA NPP validation. Sites meeting the criteria outlined by Potter et al. [26] were selected for CASA model validation. In order to meet the selection criteria, sites needed to have a minimum of 3 years of flux data and the data must span the entire year in order to compare dormant season results. Of the 51 sites reviewed, 17 sites met these criteria (with locations shown in Fig. 2) and were included in this analysis. Of the Ameriflux locations selected for validation, the majority of the sites (11 of the 17) were located in some type of forest land cover, 7 were in evergreen needle leaf forests, 3 in deciduous broadleaf forests, and 1 in mixed forest. The remaining six sites were cropland (3 sites), grassland (2 sites), and the final site was a savanna woodland site. For sites that met these criteria, Ameriflux data sets were downloaded from the Carbon Dioxide Information Analysis Center (CDIAC; http://public.ornl.gov/ameriflux/dataproducts.shtml). We selected the Level 4 gap-filled and ustar-filtered records, which contain estimated gross primary productivity (GPP) and total ecosystem respiration.

Monthly records of NPP were estimated using an uncertainty range of 40–50 % of the GPP carbon flux defined for temperate ecosystems. Waring et al. [38] evaluated a constant ratio of NPP/GPP for forested sites and found the ratio to be 0.47 ± 0.04 SD. Zhang et al. [42] found the global NPP/GPP ratio to be 0.52 with minimal variation. However, they did find densely vegetated regions to have a lower NPP/GPP ratio than sparsely vegetated regions. Comparison between a NPP/GPP ratio of 40 and 50 % and found no significant difference (p < 0.01). The NPP/GPP ratio of 0.40 had slightly better correlation than 0.50. Very little inter-annual analysis of the NPP/GPP ratio has been performed and only limited to certain specific species, however Campioli et al. [1] found inter-annual fluctuation of the NPP/GPP ratio in Beech stands to not be significant throughout the year.

Using a point intersect technique, CASA model results were extracted for the years matching the Ameriflux site data. We also extracted the neighboring 3 × 3 pixel area around the Ameriflux site to test a 1.5 × 1.5 km footprint around the validation site. The 3 × 3 pixel area was averaged to produce a single CASA model NPP value that was then compared to the Ameriflux site data. An expanded footprint was tested because air around the tower is mixing and moving due to wind and the tower measurement is indicative of the local area rather than a single point in space. Furthermore, because flux towers are typically positioned in areas surrounded by similar land cover, it is reasonable to summarize the CASA results over a local neighborhood that is less slightly variable over time than the response at a single pixel.

Next, a series of linear regressions was performed comparing Ameriflux site data with the annual CASA results. Regression analysis was performed comparing the results of the point intersection and the 3 × 3 pixel average against both the 40 % estimate of GPP and 50 % estimate of annual GPP. For revised model evaluation, seasonal CASA output was compared to the seasonal flux of the Ameriflux sites. Comparing the seasonal flux of both the ground measurements and the model estimates provides insight into how well the CASA model estimates timing of yearly productivity maxima and minima.

## Results

### Comparison of MODIS EVI values to IVI values

**Fig. 3:**
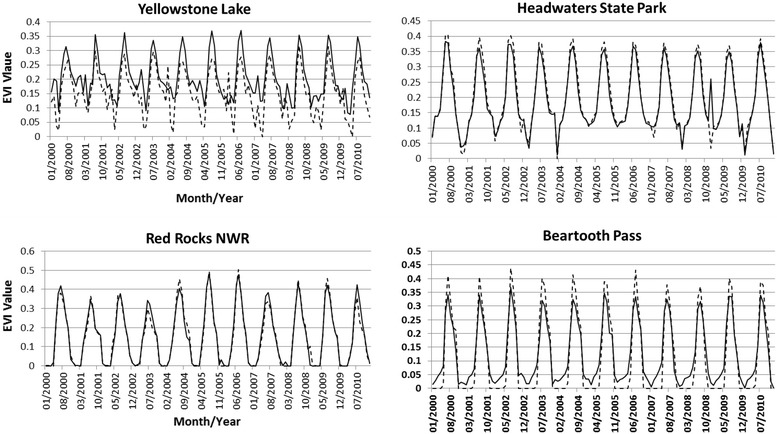
Comparison of pure-pixel interpolated EVI values and MOD13 composite EVI values for four 50-m resolution selected mountain wetland landscapes. *Solid line* is pure-pixel interpolated EVI and dashed line is MOD13 composite EVI

Four mountain wetland areas were selected to demonstrate to differences between IVI values and MOD13 composite EVI values. These locations were selected based on the authors’ previous experience working in these landscapes, and the relatively high proportion (15–53 %) of the pixel containing wetlands. Results showed a significant difference between pure-pixel EVI and MODI13 composite EVI values for three of the four sites compared (Table 1). For example, at the Yellowstone Lake location (Fig. 3), MOD13 EVI values approach zero in March and April, in contrast to the pure-pixel interpolated EVI values which had a much more muted or non-existent dip during these warming months. This difference may be a result of the pure-pixel interpolated EVI capturing annual lake level changes and vegetation greening during spring run-off periods. The remaining sites (Fig. 3) showed that the major differences between pure-pixel and MOD13 EVI occurred in the winter months of December to February, and that the pure-pixel EVI values were slightly lower in the peak summer months of July and August.

**Table 1:** Comparison of pure-pixel interpolated EVI values and MOD13 EVI values using a paired t-test

Location	Latitude	Longitude	Percent wetland (%)	t-value	df	p-value
Yellowstone Lake	44.406	−110.252	53	−13.54	132	<0.05
Headwaters State Park	45.919	−111.49	15	3.58	132	<0.05
Red Rocks NWR	44.623	−111.806	52	−1.70	132	0.09
Beartooth Pass	44.931	−109.523	30	−2.81	132	<0.05

### Combined flux tower NPP comparisons

**Fig. 4:**
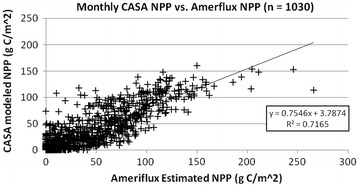
Comparison between monthly tower flux estimated NPP and monthly CASA estimates for all Ameriflux sites selected for validation

Seventeen Ameriflux towers spread across the Continental United States (CONUS) were combined to evaluate monthly NPP estimates from the modified CASA model. Using a NPP:GPP ratio of 40 % in the tower flux measurements, 1030 monthly data values plotted against the CASA model outputs resulted in an overall R^2^ of 0.72 (Fig. 4). Averaging NPP for a three cell by three cell buffer around the flux tower location resulted in a slight decrease in correlation between modeled NPP and flux tower measurements (R^2^ = 0.71). Removing flux tower sites that have been recently disturbed or managed the overall R^2^ increases to 0.82. The revised CASA monthly NPP estimates were found to be 25 % lower overall than the tower-based NPP measurements, for reasons explained below.

**Fig. 5:**
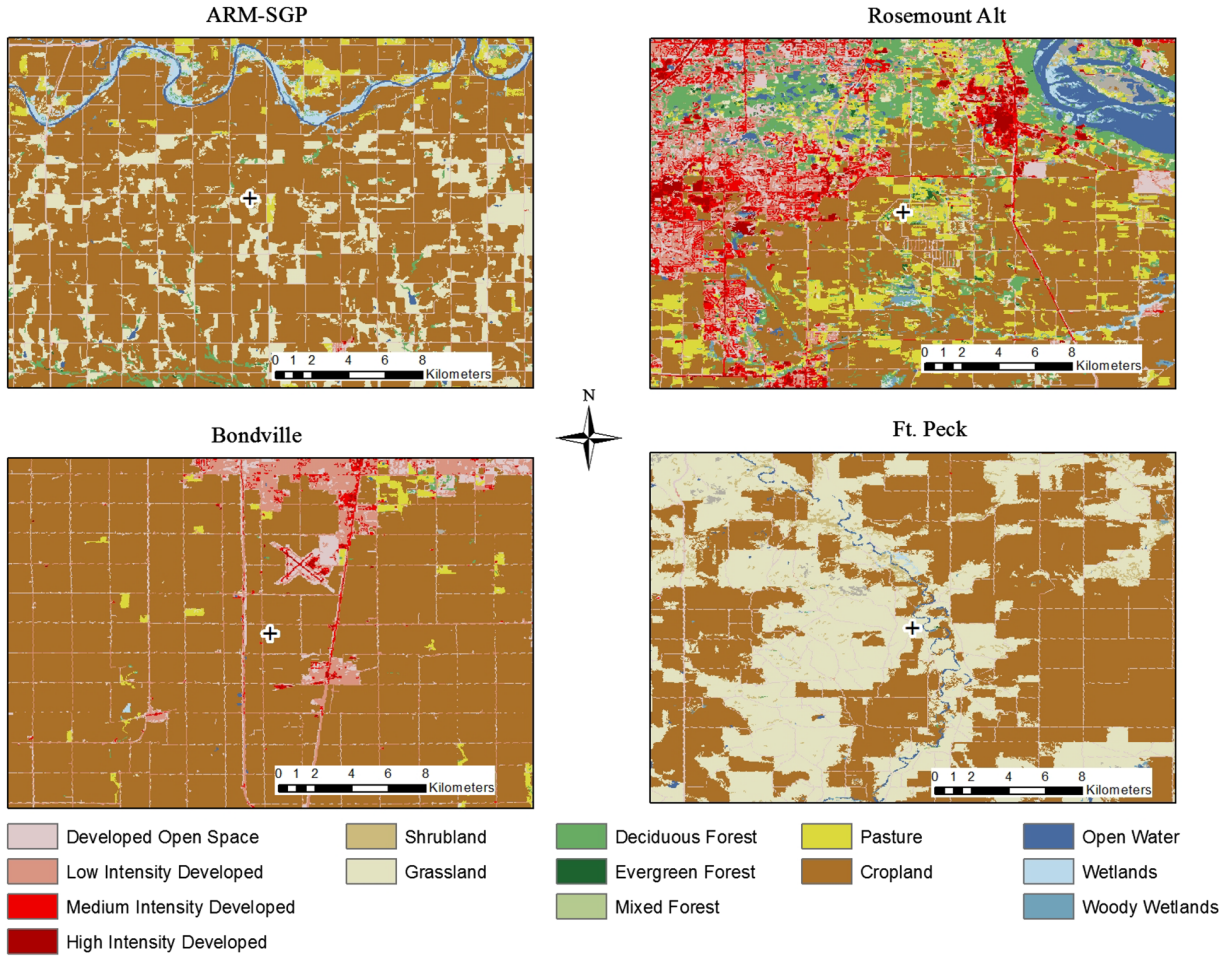
NLCD land cover maps for agricultural tower flux sites used in CASA model NPP comparisons

On a seasonal basis, the modified CASA model most closely matched the tower flux NPP during summer (R^2^ = 0.58) and autumn (R^2^ = 0.72), and most poorly in the winter (R^2^ = 0.22). Correlations were also relatively low in the spring, showing a R^2^ = 0.48. Tower sites in the Northeast and northern Midwest showed the highest levels of correlation (Table 2), with the Sylvania Wilderness flux tower in northern Michigan returning a R^2^ = 0.93 and the Morgan Monroe State Forest flux tower in Indiana a correlation of R^2^ = 0.93. Southern and western CONUS tower sites matched most poorly with CASA model estimates, with the Donaldson flux tower in Florida showing the lowest correlation of R^2^ = 0.01 and the ARM-SGP main tower in Oklahoma returning an R^2^ = 0.08. When grouped by land cover types, the combination of deciduous broadleaf forests monthly NPP measurements matched most closely with CASA model estimates, resulting a correlation of R^2^ = 0.88, followed by croplands (R^2^ = 0.73), grasslands (R^2^ = 0.65), and evergreen needleleaf forests (R^2^ = 0.57) (Table 3).

**Table 2:** CASA annual NPP validation results by Ameriflux tower site

Site	Land cover	*R* ^2^
Donaldson	Evergreen needleleaf forest	0.01
ARM-SGP	Croplands	0.08
Tonzi	Woodlands	0.23
Mize	Evergreen needleleaf forest	0.25
Blodgett	Evergreen needleleaf forest	0.34
Ft Peck	Grasslands	0.54
Goodwin Creek	Grasslands	0.54
Rosemount Alt	Croplands	0.65
Wind River	Evergreen needleleaf forest	0.70
Metolius Intermediate	Evergreen needleleaf forest	0.72
Missouri Ozark	Deciduous broadleaf forest	0.80
Bondville	Croplands	0.82
Howland West	Evergreen needleleaf forest	0.86
Howland Main	Evergreen needleleaf forest	0.86
Harvard Forest	Deciduous broadleaf forest	0.90
Morgan Monroe	Deciduous broadleaf forest	0.91
Sylvania Wilderness	Mixed forest	0.93
Overall		0.72

**Table 3:** CASA NPP validation results by land cover type within tower site

Land cover	*R* ^2^	*n*
Evergreen needleleaf forest	0.57	401
Deciduous broadleaf forest	0.88	212
Croplands	0.73	154
Grasslands	0.65	117
Woody savannas	0.23	81
Mixed forest	0.93	65

### Individual flux tower NPP comparisons

**Fig. 6:**
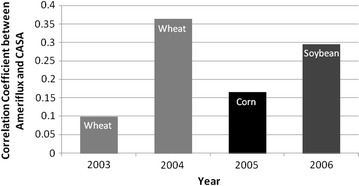
Annual correlation coefficients of measured NPP with CASA model estimates at the ARM-SGP main tower site in Oklahoma by crop rotation

The ARM-SGP site located in north-central Oklahoma showed a low correlation coefficient between flux tower NPP measurements and CASA model estimates (Fig. 6). This site (and several others shown in Fig. 5) was located in a region dominated by a mix of cropland and grassland cover types, which at ARM-SGP consisted of wheat (*Triticum aestuvum* L.), corn (*Zea Mays* L.) and soybean (*Glycine Willd.*). During flux measurements from January 2003 through October 2006, multiple rotations of different crops occurred. During 2003–2004, common wheat was planted, in 2005 corn was planted instead of wheat, and soybeans were planted in 2006. Comparing monthly average NPP for each separate year, 2003 showed an R^2^ = 0.1, 2004 showed an R^2^ = 0.36, 2005 showed an R^2^ = 0.17, and 2006 showed an R^2^ = 0.30.

**Fig. 7:**
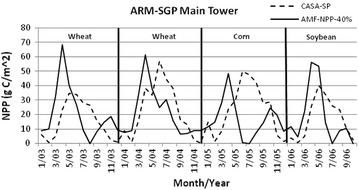
Comparisons of monthly NPP flux in cropland types at the ARM-SGP main tower in Oklahoma

A summary of the monthly flux comparisons (Fig. 7) revealed that the CASA model does not yet account for either irrigation schedules that boost NPP of crops as intended by the farmers, or for crop harvests that are typically occurring in June–August of each year. This lack of sensitivity to crop-specific irrigation likely explains the low monthly NPP correlation observed at this site as well as the 25 % underestimation of NPP shown for all tower sites combined in Fig. 4. There was also a consistent increase in tower-measured NPP flux in September and October for most crops at the ARM-SGP site, which was not estimated by the CASA model during these cooler months outside the main corn and soybean growing seasons.

**Fig. 8:**
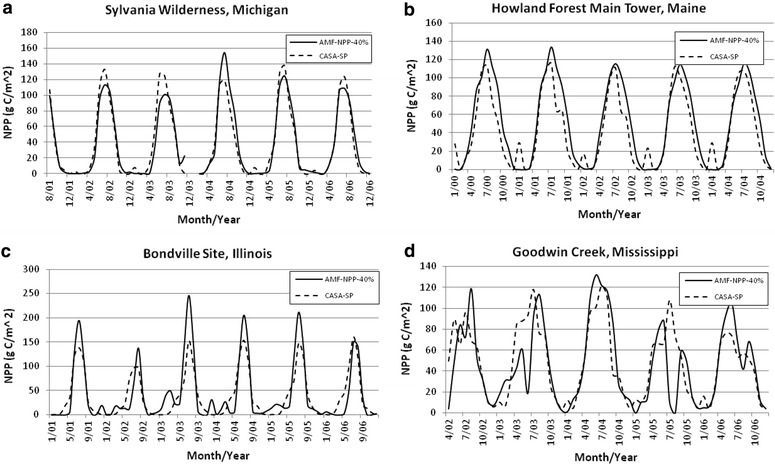
Comparison of monthly NPP patterns between Ameriflux measured NPP and CASA modeled NPP. **a** Sylvania Wilderness, Michigan, mixed forest land cover, **b** Howland Forest Main Tower, Maine, evergreen needleleaf forest, **c** Bondville, Illinois, cropland, **d** Goodwin Creek, Mississippi, grassland

Four example Ameriflux sites (two forests, one cropland, and one grassland) where the CASA model NPP matched closely with tower-measured monthly NPP (Fig. 8) resulted in R^2^ values between 0.82 and 0.93. The Goodwin Creek grassland sight (R^2^ of 0.54), however, was an exception to this pattern. In most years, the revised CASA model closely tracked the seasonality of NPP and the peak summer NPP values in these tower flux measurements. It is worth noting that the Bondville tower site had the lowest level of mixed land cover types (with surrounding grasslands and pastures) of any of the cropland locations shown in Fig. 5.

The seasonal pattern in tower-measured NPP at Goodwin Creek grasslands was inconsistent from year-to-year (Fig. 8), which differed from the CASA model NPP, which suggested some management or local disturbance impacts on the tower fluxes that are not unaccounted for by the CASA model. The Ameriflux data source (available at http://ameriflux.ornl.gov/) states that grass surrounding the base of the tower was mowed periodically to maintain a height consistent with the regional grasslands, which confirms the sudden and unpredictable loss of tower-measured NPP at this location.

**Fig. 9:**
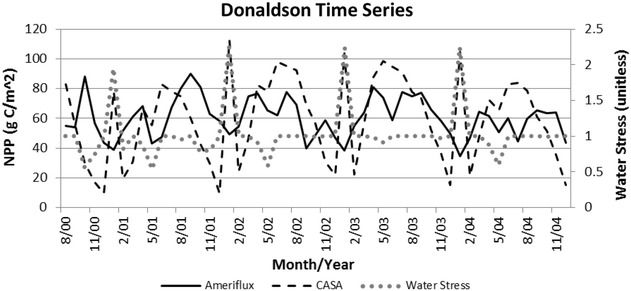
Monthly NPP comparisons at the Donaldson tower site in Florida

Another site that showed a low correlation with CASA estimated NPP was the Donaldson flux tower in Florida. This site is a managed slash pine (*Pinus elliottii*) plantation ecosystem in north-central Florida [29]. The stand is even-aged with the overstory comprising of 100 % slash pine with assorted native species in the understory. Review of the measured NPP pattern shows consistent CASA mode overestimation in January. This is likely a result of the site being located in a warm climate, where evapotranspiration remains high all year but precipitation is inconsistent, leading to spikes that result from rapid soil wetting and drying. Graphing the soil water scalar term against NPP (Fig. 9) shows spikes in soil water stress echoed within CASA NPP. Management and disturbance also played a role in the poor result at the Donaldson site. A 100-year drought was reported from 2000 through the summer of 2002. The CASA algorithm was able to detect this drought as indicated by the similar amplitude observed in the NPP time-series flux; however, CASA was not able to match peaks and troughs measured at the site. Following the drought, fertilizer was applied in 2002 to the plantation. Interestingly, CASA slightly over-predicted the tower NPP measurements despite the fertilizer application, and did not detect a large decline in productivity in June 2002. No major disturbances were reported during 2003 and this year resulted in the highest correlation between the CASA-modeled and the measured NPP fluxes. A series of tropical storms struck the site during the summer of 2004, but the CASA algorithm did not detect the loss of productivity of the forest overstory due to the site being inundated in September following this series of storms.

## Discussion

Generally, we found that the revised CASA model with pure-pixel interpolated EVI performed well at tower sites where vegetation was not manipulated or managed and had not been recently disturbed. Sites such as the Sylvania Wilderness and Morgan Monroe, which are large, relatively undisturbed tracts of temperate forest, both had very high correlations with CASA-estimated NPP. Sites such as Goodwin Creek grasslands and the Donaldson managed pine plantation locations both had relatively low correlations with CASA-estimated NPP, which we attribute to the disturbances experienced by these sites.

Cropland-dominated tower site comparisons exhibited several instances of mistiming and underestimation of peak monthly NPP by the revised CASA model. The southern Plains states fluxes typically measured a cropland NPP peak in April-May, followed by a steep decline in NPP in June-August, which presumably result from, respectively, early springtime rainfall and irrigation and mid-summer harvesting of the various rotating crops. The CASA model did not include the effects of irrigation on boosting crop NPP and yield, which is very commonly practiced in farmlands of the Plains states [28]. The CASA model did not detect a regular June-August decline in NPP measured due to crop harvest practices, indicating the pure-pixel interpolated EVI may have remained high in summer due to inclusion of surrounding grassland and pasture areas that were not harvested at that time and were shown to surround most central tower locations in Fig. 5.

Crop rotation also played a role at some agricultural tower sites. At the ARM-SGP tower site in 2005, corn was planted instead of wheat, and the change from a C3 plant to a C4 plant may explain the drop in correlation with the CASA model estimates, since the CASA algorithm uses a constant light-use efficiency term. However, C4 plants (such as corn) tend to have higher light-use efficiency terms than C3 plants (such as wheat). Soybeans were planted in 2006 and again we observed a slight increase in correlation in the rotation from a C4 crop to a C3 crop. This shortcoming in the CASA model could be mitigated by using different light-use efficiency terms based on the crop type found at a particular location. This is one technique that Yu et al. [41] used to modify the CASA model to estimate productivity in China.

Changes and discrepancies in land cover used in the study also explain some of the variation in the results. This model relies heavily on land cover inputs and the broad cover classes used may not capture regional differences in vegetation. For example, evergreen forests of the Pacific Northwest are not comprised of the same species as the Slash Pine forests in Florida yet the model treats these land covers as the same. Additionally, the model also assumes that land cover is consistent year to year, which is not what is happening in reality. The static nature of land cover limits the ability of the model to capture land cover changes and adjust NPP estimates accordingly. This can be observed by the low R^2^ achieved at sites with crop rotations.

Previous comparison studied by Li et al. [13] concluded that variations in NPP in woodland locations of drought-prone climate zones, such as the central California Tonzi Ranch tower site, cannot be matched closely by CASA unless soil water availability was modified in the model structure. This site was a savanna consisting of scattered blue oak trees (*Quercus douglasii*), with occasional gray pine trees (*Pinus sabiniana* L.), surrounded by grazed grassland. Oak trees in this region were able to continue to transpire into the summer months, albeit at low rates, under very dry soil conditions and maintain basal levels of carbon metabolism, because tree roots were able to access sources of water in the soil unavailable to grass roots. Consequently, for CASA model applications in oak woodlands of California, an adjustment should be in the available water storage content for the deeper rooting layer of shrubs and trees that may be present at such sites. This adjustment made available 80% more soil water for transpiration by shrubs and trees than is commonly set for other moist forested climate zones of the western United States, and resulted in an R^2^ match of 0.89 between monthly tower measurements and CASA estimated NPP.

## Conclusions

Using image fusion of Landsat and MODIS satellite data products to enhance the CASA ecosystem model shows promise for accurate monthly NPP estimation, especially in heterogeneous but relatively undisturbed landscapes. However, more validation and work will be required to fully understand how well the CASA model can perform at managed cropland sites. This is due to the fact that the model inputs and algorithms are not yet sensitive to some management practices, such as irrigation and crop rotations. Further validation in wetlands and mountainous landscapes with new tower flux measurements will be required to fully document the advantages of image fusion to improve model NPP estimates.

## Abbreviations

AVHRR: Advanced Very High Resolution Radiometer
CASA: Carnegie Ames Stanford Approach
CDIAC: Carbon Dioxide Information Analysis Center
CONUS: Continental United States
eMax: light utilization efficiency term
EVI: Enhanced Vegetation Index
GLDAS: Global Land Data Assimilation System
GPP: gross primary productivity
IVI: interpolated vegetation index
NARR: North American Regional Reanalysis
NCEP: National Centers for Environmental Prediction
NLCD: National Land Cover Database
NPP: net primary productivity
